# Bitter Perception and Effects of Foods Rich in Bitter Compounds on Human Health: A Comprehensive Review

**DOI:** 10.3390/foods13233747

**Published:** 2024-11-22

**Authors:** Kaina Qiao, Mingxia Zhao, Yan Huang, Li Liang, Yuyu Zhang

**Affiliations:** 1 Food Laboratory of Zhongyuan · Beijing Technology and Business University, Luohe 462300, China; 2 Key Laboratory of Geriatric Nutrition and Health, Ministry of Education, Beijing Technology and Business University, Beijing 100048, China; 3 Key Laboratory of Flavor Science of China General Chamber of Commerce, Beijing Technology and Business University, Beijing 100048, China; 4 Food Laboratory of Zhongyuan · Luohe Food Engineering Vocational University, Luohe 462300, China

**Keywords:** bitter taste, bitter perception, bitter compounds, health benefits

## Abstract

Bitter food, because of its unique taste, is not popular with the public, and is even considered to be difficult to swallow. By binding to specific sites of bitter receptors (26 hTAS2Rs), bitter compounds activate the downstream signaling pathways mediated by G protein, which convert chemical signals into electrical signals that are ultimately transmitted to the brain to produce the bitter perception. The intensity of bitterness is mainly determined by the hydrophobic recognition region of bitter receptors. The bitter compounds in foods mainly include alkaloids, polyphenols, terpenoids, amino acids, etc. Foods rich in bitter taste are mostly natural such as beans, nuts, and coffee, etc. Studies have proven that bitter foods have biological activities such as preventing hyperlipidemia, hypertension, hyperglycemia, anti-inflammatory, antitumor, antibacterial, antioxidant, and exhibit neuroprotective effects and other biological activities. The purpose of this review is to explore the bitter perception and the biological activity of bitter compounds, clarify the mechanism of their action on human health, and provide theoretical guidance for the development and application of functional foods.

## 1. Introduction

Bitter compounds have specific functional groups and molecular structures that allow them to interact with bitter receptors in human taste buds, triggering the perception of a bitter taste. In addition, bitter compounds tend to have a lower solubility and higher volatility, which provide them with a unique distribution and release pattern in foods. For example, tea, coffee, and cocoa have significantly reduced bitter intensity at higher temperatures, so high temperatures may reduce bitterness [1]. Moreover, the bitterness is characterized by its interaction with other flavors. Specifically, bitterness has an inhibitory effect on sweet and salty tastes, which requires special attention to the balance of bitterness when seasoning. At the same time, some bitter compounds have a synergistic effect with sour compounds, which can enhance the overall taste experience. The intensity of bitterness is mainly determined by the hydrophobic recognition region of the bitter receptors. By binding to specific sites of bitter taste receptors (26 hTAS2Rs), bitter compounds activate the downstream signaling pathways mediated by G protein, which convert chemical signals into electrical signals and finally transmit them to the brain to produce the bitter perception [2].

Bitter compounds in food are unique ingredients that are widely present in a variety of ingredients. These bitter compounds mainly include alkaloids, polyphenols, terpenoids, and amino acids. Alkaloids are a class of compounds with an obvious bitter taste and are widely found in many natural foods. For example, coffee beans and tea are rich in alkaloids such as theophylline and theoblaine [3]. Polyphenols are another important class of bitter compounds that are widely distributed in many fruits, vegetables, and red wine. For example, the proanthocyanidin in grapes and resveratrol in red wine are representative substances of polyphenols. Terpenoids are also a common bitter ingredient that play important roles in many natural foods. For instance, limonene, which exists in citrus fruits, is a typical terpenoid compound that gives these fruits a unique aroma and bitter taste [4]. In addition, amino acids are also an important source of the bitter taste in food products. Some sulfur-containing amino acids such as methionine and cystine as well as some aromatic amino acids, such as phenylalanine and tyrosine, all have a certain bitter taste [5]. Beans are one of the most important sources of bitter compounds, such as black beans and mung beans, and contain a variety of bitter ingredients (polyphenols). Nuts are also bitter foods; walnuts, almonds, and hazelnuts are rich in alkaloids and polyphenols, which give nuts their unique taste and aroma [6].

Foods rich in bitter compounds not only bring a unique experience to the body’s taste buds, but are also beneficial to human health. In recent years, studies have found that bitter foods have significant effects on regulating blood sugar levels; specifically, bitter foods have the effect of lowering blood sugar levels, increasing serum insulin levels, improving insulin sensitivity, maintaining blood sugar levels, lowering cholesterol, increasing high-density lipoprotein, and reducing low-density lipoprotein and triglycerides. In addition, bitter foods have an obvious antihypertensive effect. Bitter foods can also inhibit the production of inflammatory factors, reduce inflammatory responses, clear free radicals in the human body, and reduce the oxidative stress responses, thus helping to prevent the occurrence of many chronic diseases. Moreover, bitter foods can inhibit lung cancer by inhibiting EGFR, and inhibit tumor spheres through mitochondrial superoxide production and mitochondrial debris organization, leading to tumor cell apoptosis. Bitter foods can bind to the cell membrane or cell wall of bacteria, destroy their integrity, and lead to bacterial death, thus exerting an antibacterial effect. The action mechanism of some bitter compounds, such as caffeine, on neuroprotection is a multilevel, multipathway process involving multiple aspects including signal transduction, apoptosis, neurotransmitter receptors, and ion channels [7,8].

This review, by summarizing the bitter perception mechanism, bitter compounds, and the application of foods rich in bitter compounds in the food industry, elucidates the sensing mechanism of bitter taste as well as the therapeutic role of bitterness on chronic diseases. Therefore, by summarizing foods rich in bitter compounds and their effects on human health, this paper presents an idea of how to retain the health value of bitter foods while reducing the human bitter taste perception, which provides a theoretical basis for the subsequent study of bitter compounds.

## 2. The Perception Mechanism of Bitterness

### 2.1. Bitter Receptors

The mechanism of bitterness perception is the key to bitterness recognition. This mechanism is mainly accomplished by the human taste system, especially the bitter receptors in the mouth, which are the G protein-coupled receptors that are mainly distributed on the surface of the tongue and the cells of the throat, and are recognized by the hTAS2Rs receptor [2]. Bitter receptors hTAS2Rs are generally thought to be monomers, but recent research has shown that they can also form homodimers and heterodimers. The hTAS2Rs family in rodents has more than 40 members, and there are 26 functional genes encoding hTAS2Rs in humans. hTAS2R1, hTAS2R3, hTAS2R4, hTAS2R14, and hTAS2R16 have been reported to be able to identify bitter peptides, hTAS2R1 is specific for bitter dipeptides and tripeptides, hTAS2R3 can only sense chloroquine (94 different natural and synthetic compounds were tested), and hTAS2R14 is able to sense at least 33 compounds [9,10]. However, one single bitter substance can often activate a variety of different hTAS2Rs, such as quinine, which activates nine different human hTAS2Rs receptors [11]. Studies have shown that hTAS2R38 recognizes the N-C=S group, and hTAS2R16 mainly recognizes the exogenous compounds. The interaction between the bitter substance and the receptors generates a second signal that activates a series of physiological responses downstream [12].

### 2.2. Bitter Signal Delivery Pathway

**Figure 1:**
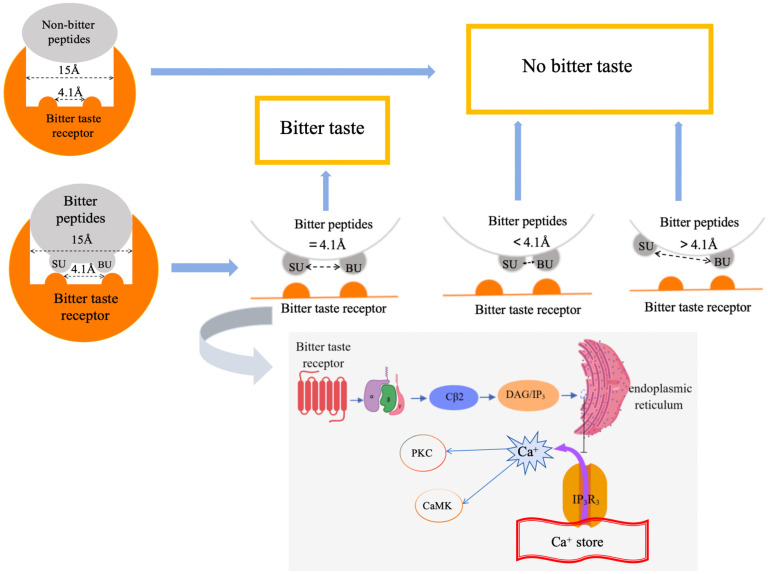
Mechanism of bitter taste conduction in humans.

The mechanism by which bitter peptides bind to bitter receptors was first proposed by Ishibashi et al. A schematic diagram of the proposed mechanism is shown in Figure 1. Bitter taste receptor signaling pathways are diverse, and it has been reported that bitter peptides have two functional units: the “binding unit” (BU) and the “stimulating unit” (SU). The hydrophobic side chain of bitter peptides can provide BU to bitter receptors, while SU consists of a large basic group or hydrophobic group that acts as the determining site of bitterness [13]. The two units are evenly spaced (about 4.1 Å), while the “pocket” size of the bitter receptor is estimated to be 15 Å. The perception of bitterness initiates the binding of bitter peptides to bitter receptors, thereby inducing a neural signaling cascade. In addition, the bitterness intensity is mainly determined by the hydrophobic recognition region of bitter receptors. The Gβγ subunit plays a key role in the regulation of taste signaling pathways. Once the T1Rs and hTAS2Rs receptors are activated by the corresponding taste substances, the Gβγ dimer is released from the G protein complex and interacts with its downstream effector molecules [14]. One of them is phospholipase Cβ2, which is activated in response to Gβγ dimers, which in turn hydrolyzes membrane phospholipids to produce diacylglycerol (DAG) and inositol triphosphate (IP3). DAG and IP3 further regulate the intracellular signal transduction processes in the cell. Of particular note is the ability of IP3 to drive to open the Ca^2+^ channel on the endoplasmic reticulum (ER), resulting in elevated cytosolic Ca^2+^ levels. Changes in Ca^2+^ concentration not only activate kinases such as protein kinase C (PKC) and calmodulin-dependent protein kinase (CaMK), but also affect the activity of several taste-related ion channels and transporters (Figure 1) [15]. These kinases and ion channels play an indispensable role in the transmission, amplification, and integration of taste signals. Moreover, the cAMP signaling pathway also plays an important role in taste signal transduction. Although the Gα subunit is capable of activating cAMP signaling, studies have shown that the Gβγ subunit is equally significant in regulating this pathway. The production and accumulation of cAMP can activate protein kinase A (PKA), which then regulates the phosphorylation state of a series of taste-related proteins to realize the long-term regulation of taste signals [16].

### 2.3. Bitterness Perception and Health

When an individual ingests a food rich in bitter compounds, hTAS2R responds by triggering the neural pathways that lead to the bitter perception. In addition, the bitter perception also affects human health by influencing individual preference driving behaviors such as dietary choices, alcohol, and tobacco intake [17]. In other words, aversion driven by bitterness can lead to the individual’s rejection of other nutrients in ingested foods, for example, bitter melon and citrus fruits. These are often bitter but non-toxic, and the rejection of these foods can lead to a reduced intake of vitamins and other nutrients. Legumes and cruciferous vegetables contain compounds that cause hTAS2Rs reactions such as flavonols and other glucosides [18], and the intake of these foods is beneficial. Some studies have illustrated that hTAS2Rs, which is activated by bitter compounds, may mediate a protective response to the excessive intake of toxic substances and may serve as a new drug target for the treatment of hypothyroidism or hyperthyroidism [19]. Furthermore, hTAS2R agonists may act directly on immune cells to exert their anti-inflammatory effects [20].

## 3. Bitter Compounds

### 3.1. Alkaloids

**Figure 2:**
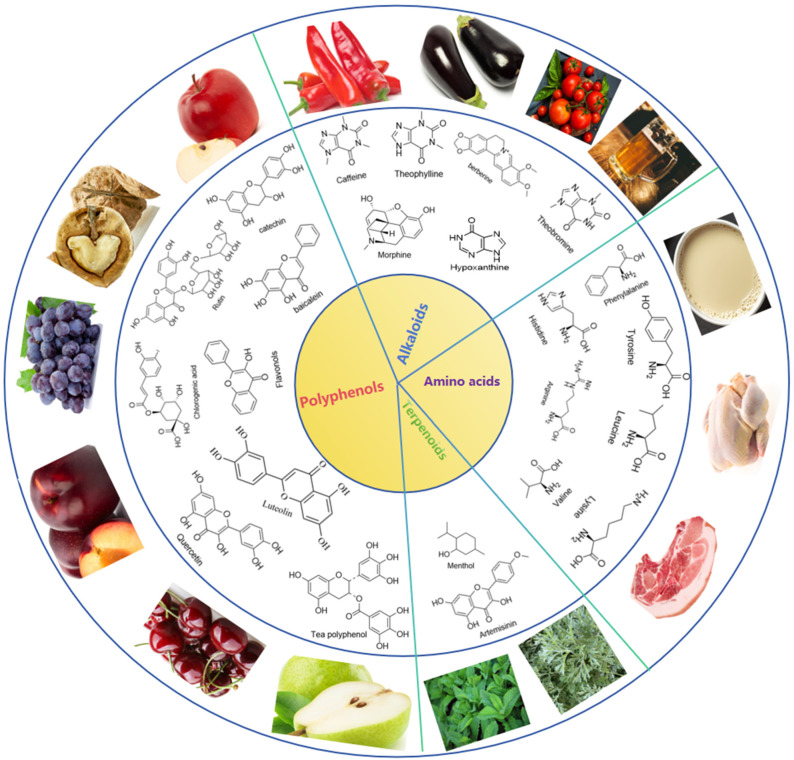
Bitter compounds.

Alkaloid compounds (Figure 2) are a class of natural compounds with a bitter taste that widely exist in nature [21] and usually have complex chemical structures and diverse biological activities, mainly containing basic nitrogen atoms. These compounds play an important physiological role in plants and are widely used in medicine, pesticides, and foods. Alkaloid bitter compounds include caffeine, theobroline, morphine, berberine, and hypoxanthine. Caffeine is an alkaloid widely found in coffee, tea, chocolate, and certain soft drinks, and the sources of dietary coffee in people’s daily life are shown in Table 1 [22,23]. Caffeine has a central nervous system stimulant effect and is often used as a pick-me-up [24,25]. Theophylline is an alkaloid that exists naturally in a variety of plants. Its main sources include tea (especially green tea, black tea and oolong tea), coffee beans, cocoa beans, and some tropical plants. Coffee beans with a higher caffeine content, such as Arabica beans and Robusta beans, are rich in theophylline. The content of theophylline varies according to the variety, origin, and degree of baking. In general, less roasted coffee beans have higher theophylline [26]. Cocoa beans contain less theophylline content than tea leaves and coffee beans [27]. Tropical plants such as Guarana and Matea also contain theophylline, which are often used to make energy drinks and tea drinks [28]. Dark chocolate contains a higher percentage of cocoa solids with a high theobromine content. Dark chocolate with a high cocoa content (usually over 70%) should be chosen to enjoy the rich chocolate flavor and health benefits of theobromine [29]. Different types of tea, such as green, black, and oolong teas, have slightly different levels of theobromine [30]. Theobromine is found in coffee beans, which is one of the reasons why coffee has a bitter taste and refreshing effect. Although the main active ingredient in coffee is caffeine, theobromine also contributes to the overall flavor of coffee [31]. Guarana is a tropical vine with abundant cacobine in its seeds and fruits and is often used to make energy drinks and weight-loss products for its refreshing and metabolic effects [32]. Madtea is a tea drink widely popular in South America with theobromine in its leaves, and has the effect of refreshing the brain and enhancing physical strength [28]. The seeds of cocoa fruit contain theobromine, which is often used for making carbonated drinks such as cola. Morphine has a unique bitter taste. This bitter taste originates from certain chemical structures in the morphine molecules, especially from the presence of its phenolic hydroxyl groups and nitrogen atoms. Poppy seeds are the seeds of poppy plants, commonly used in baked goods, and they may contain trace amounts of morphine. The use of poppy seeds as a food additive is legal in some countries, but its content must be strictly controlled [33]. Some fruits and vegetables also contain trace amounts of morphine such as immature tomatoes and eggplant [34]. These levels are usually very low and cannot have significant effects on the human body. Although the aforementioned foods contain trace amounts of morphine, they are usually insufficient to produce significant pharmacological effects. Attention may only be raised in specific situations, for example, certain diseases or excessive intake. Hypoxanthine is a purine alkaloid that is widely found in a variety of foods including animal viscera, seafood, beer, etc. Consuming foods (animal viscera, hotpot seasoning, seafood) that contain high levels of hypoxanthine can exacerbate the condition in people with gout or those at risk of kidney stones. Although the hypoxanthine content in beer is relatively low, frequent drinking has adverse effects on uric acid levels [35]. However, some studies have pointed out that it has a positive effect on the human body under moderate intake. Because purine compounds are natural products of cellular metabolism, they are involved in the generation and transmission of energy. In addition to the common alkaloid bitter compounds described above, there are many other alkaloid bitter compounds with different chemical structures and bioactivity such as quinine. These compounds have important applications in the medical field such as treating cancer, cardiovascular diseases, and neurological diseases, etc. At the same time, some alkaloid bitter compounds also have certain pesticide activity and can be used to control crop diseases and pests [36].

### 3.2. Polyphenols

Polyphenolic bitter compounds exist widely in nature, especially in the plant kingdom. They not only give many plants a unique bitter taste, but they also have a variety of biological activities such as antioxidant, anti-inflammatory, antibacterial, and anticancer [37]. The structure of polyphenolic compounds is diverse including chlorogenic acid, rutin, flavonol, yellow zen, anthocyanins, quercetin, catechin, and so on [38].

Chlorogenic acid is a widespread polyphenolic compound found in plants with various biological activities including antioxidant, anti-inflammatory, and antimicrobial activity. In addition to green tea and coffee, there are many other foods rich in chlorogenic acid. Chlorogenic acid in apples helps lower blood sugar and cholesterol levels [39]. Sunflower seeds are rich in chlorogenic acid, which has a strong antioxidant effect. The flesh and skin of pears contain chlorogenic acid, and the intake of pears can boost immunity and prevent colds and flu. Both the leaves and roots of chicory contain chlorogenic acid, which helps to improve digestive system function and has certain anti-inflammatory effects [40]. Olive oil contain chlorogenic acid, which is an important component of the Mediterranean diet that helps reduce the risk of cardiovascular disease [41]. Mint tea or fresh mint leaves contain chlorogenic acid, which has the effect of refreshing and relieving stomach flatulence. Peanuts are not only rich in healthy fat and protein, but also contain a certain amount of chlorogenic acid, and a moderate consumption of peanuts can help maintain cardiovascular health [42].

Rutin is a polyphenolic compound widely found in nature and has attracted much attention for its outstanding antioxidant and anti-inflammatory properties. Rutin not only has significant pharmacological activity, but has also been widely used in the food industry because of its unique flavor and color. Onions are rich in rutin, and their regular consumption can help lower blood pressure and improve cardiovascular health [43]. Green tea is rich in rutin, and the frequent consumption of green tea helps with antioxidants, as an anti-inflammatory, and reduces the risk of multiple chronic diseases. Apples are rich in rutin and many other beneficial ingredients, which can help to improve intestinal health and reduce the risk of cardiovascular disease [44]. Grapefruit is not only rich in rutin, but also vitamin C and fiber, which help lower cholesterol levels and improve cardiovascular health. Red chili peppers are rich in rutin, which helps boost metabolism and reduce the risk of certain cancers [45]. Barbary wolfberry also contains rutin, adding a unique flavor and nutrition to the food [46].

**Figure 3:**
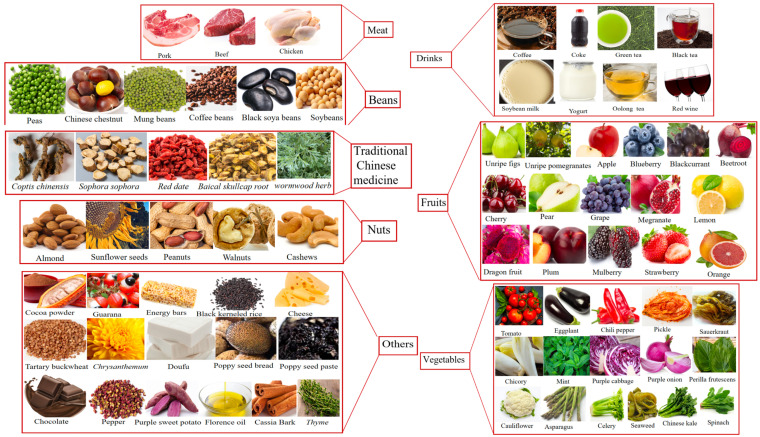
Representative foods rich in bitter compounds.

Some flavonol-rich foods, such as onions and garlic, can help reduce the risk of cardiovascular disease (Figure 3). Berry fruits such as blueberries, blackberries, and strawberries and citrus fruits such as oranges, grapefruit, lemons and limes contain flavonols [47]. Many vegetables also contain flavonols such as broccoli, spinach, and asparagus. Red wine and dark chocolate also contain flavonols. Soybeans, black beans, and red beans [48] as well as soy products such as tofu and soybean milk are not only high-quality plant protein sources, but also contain considerable flavonols. Whole grains such as oats, brown rice, and quinoa also contain flavonols [49].

Baicalein is a natural compound with various health benefits, mainly found in some foods and Chinese medicinal herbs. The root of scutellaria baicalensis is rich in baicalin, which has anti-inflammatory and antiviral effects [50].The seeds of Tartary buckwheat contain baicalin, which has the effect of lowering blood sugar and blood lipids [51]. Chrysanthemum tea has the effect of clearing heat and detoxifying eyes, and regular drinking can help relieve eye inflammation [52]. In addition, some dark green vegetables such as spinach and kale contain trace amounts of baicalein [53].

Anthocyanins are a natural pigment widely found in various plants. In addition to berry foods such as blueberries, blackberries, and purple grapes, many other foods are also rich in anthocyanins [54] such as the flesh of dragon fruit, purple skin of eggplant, purple sweet potato, and mulberry [55,56]. The purple skin of purple onions also contains a certain amount of anthocyanins [57,58].

Onion is one of the important sources of quercetin, especially red onion, which has a high quercetin content [59]. Citrus fruits such as orange, grapefruit, lemon, and other fruits contain quercetin in their skin and pulp [60]. Broccoli and olive oil also contain quercetin [61]. Red wine in the fermentation process will produce quercetin; in addition to red wine, grapes themselves also contain quercetin, especially in grape skin and grape seed [62].

Luteolin exists in a variety of plants and has a variety of pharmacological activities such as anti-inflammatory, antitumor, antibacterial, antiviral, etc. In clinical practice, they are mainly used for cough, expectorant, anti-inflammatory, uric acid, hepatitis and so on. Luteolin was originally isolated from the leaves, stems, and branches of luxocolin, and then luteolin was also found in vegetables such as cabbage, beets, broccoli, carrots, and celery [63].

Catechins are a class of polyphenolic compounds widespread in plants and have multiple health benefits. Aside from green tea and black tea [64], many other foods are also rich in catechins. High cocoa dark chocolate is rich in catechin. A dark chocolate with at least 70% cocoa should be chosen to enjoy the health benefits of catechins [65]. The skin and pulp of apples contain a higher level of catechin. High levels of catechins have been found in blueberries, cherries, walnuts, red wine, olive oil, cranberries, pomegranate, purple cabbage, peanuts, black beans, red beans, and tofu [66,67].

### 3.3. Terpenoids

Terpenoid bitter compounds play an important role in medicine, food, agriculture, and other fields. Naringenin is a typical terpenoid bitter compound, mainly derived from citron. In addition to citron, other citrus fruits such as orange, lemon, lime, and citrus also contain naringin, especially in the peel part, which is richer in naringenin [68]. Green tea and oolong tea also contain naringenin. Honey, collected from the nectar of citrus plants, also contains naringenin [69]. Naringenin has a strong bitter taste and unique aroma, and is often used as a food additive [5]. In addition, naringenin also has certain pharmacological effects, such as antioxidant and anti-inflammatory, which are beneficial to human health [70]. Artemisinin is extracted from *Artemisia annua*, and *Artemisia annua* tea is a traditional drink made from the young leaves and flowers of *Artemisia annua* [71]. Drinking Artemisia tea can help prevent malaria, boost immunity, and relieve fatigue. Menthol, a common terpene bitter compound, is mainly found in peppermint, and is widely used in food and daily chemical products such as chewing gum, toothpaste, and beverages [72]. In addition, menthol also has certain pharmacological effects such as headache and skin itching [73].

### 3.4. Amino Acids

Bitter amino acids come from a wide range of sources and exist in natural products as well as processed foods. Studies have shown that a bitter taste is the result of the binding of hydrophobic amino acids to bitter receptors. Some hydrophobic amino acids, such as histidine (His), arginine (Arg), tyrosine (Tyr), valine (Val), phenylalanine (Phe), lysine (Lys), and leucine (Leu), have a bitter taste [74], among which His, Val, Phe, Lys, and Leu are also essential amino acids with important physiological functions in the human body. By analyzing the bitter intensity of the hydrophobic amino acids in the protein solution, the results showed that the higher the content of hydrophobic amino acids, the greater the bitter intensity [75]. The protein itself does not have a bitter quality or has a weak bitter taste, but the bitter taste becomes obvious after protease hydrolysis. This is mainly due to the fact that inside the proteins, the side chains of most hydrophobic amino acids fail to make contact with the bitter receptors, and therefore do not produce a bitter taste. However, these originally internal hydrophobic amino acid side chains are gradually released into the solution during the hydrolysis of proteases, and with the gradual deepening of hydrolysis, the release of hydrophobic amino acids correspondingly increases, thus increasing the intensity of the bitter taste [76].

His comes from a variety of foods including meat, seafood, legumes, nuts, seeds, and dairy products. His is an essential amino acid that maintains normal metabolism, growth, and development of the human body. Among the vegetables, peas and seaweed are good sources of Arg. Milk, yogurt, cheese, and other dairy products also contain a certain amount of Arg. Arg has antioxidant and anti-inflammatory effects [77]. Beef, pork, and chicken are all rich in Tyr [78], and walnuts, almonds, cashews, and other nuts also contain Tyr, which has the effect of reducing blood fat [79]. Val is one of the eight essential amino acids for the human body, which can promote normal growth of the body, repair tissues, regulate blood sugar, and other functions. Dairy products, grains, and legumes are high-quality sources of Val [80]. Beef, mutton, and pork, tofu, soy milk, and soybeans are high in Phe, which has a cholesterol-lowering effect. Soybeans, black beans, peas, broad beans, and other beans are rich in Lys. Nuts such as almonds, walnuts, peanuts, and cashew nuts also have a relatively high content of Lys. Studies have shown that Lys has antioxidant and anti-inflammatory effects [81]. Brown rice, wheat germ, peanuts. and walnuts are important sources of Leu, which has various health care functions such as antioxidant and cholesterol reduction [82].

## 4. Health Benefits of Bitter Compounds on the Human Body

### 4.1. Prevent Hyperlipidemia, High Blood Pressure, and High Blood Sugar

Bitter gourd is a kind of food rich in bitter compounds, and its main bitter components are momordicine I and momordicine II. Recent studies have shown that its extract can improve the situation of centripetal obesity caused by high-cholesterol, high-fat diet, and excessive fat content in the blood [83]. Momordica II could significantly stimulate insulin secretion at concentrations of 10 and 25 μg/mL in MIN6 β-cells. Studies have also found that adding a moderate amount of bitter melon to the daily diet could reduce the rate of weight gain and the fat mass of viscera as well as have hypoglycemic and anti-diabetic effects, which may be due to the increased fatty acid oxidation levels [84]. Cocoa tea is rich in bitter compounds such as theobromine, and an aqueous extract of cocoa tea had a significant and moderate hypotensive effect. Theobromine regulates the expression of PPARγ and C/EBPα through AMPK and ERK/JNK signaling pathways in 3T3-L1 preadipocytes, thereby inhibiting adipocyte differentiation and further inhibiting adipocyte formation and reducing the occurrence of obesity [85]. Unlike the blood pressure fluctuations and drug dependence problems that may be brought about by some antihypertensive drugs, the antihypertensive effect provided by cocoa tea is lasting and stable. Further studies have also found that the antihypertensive mechanism of cocoa tea and traditional tea mainly occurs in the peripheral sites, but not in the central nervous system [86,87,88].

**Figure 4:**
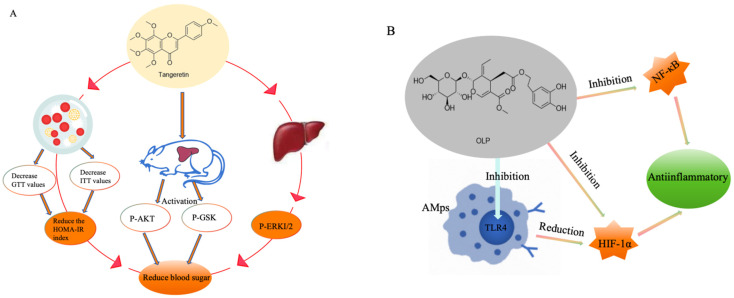

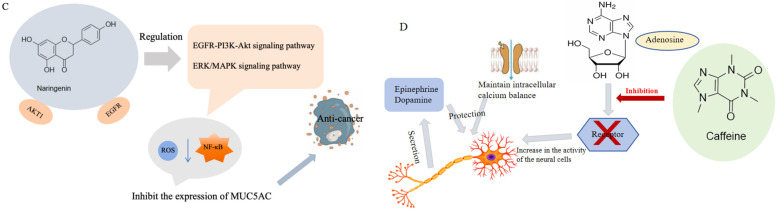
The mechanisms for the health benefits of bitter compounds on the human body. (**A**) Hypoglycemic mechanism of tangeretin, (**B**) anti-inflammatory mechanism of OLP, (**C**) antitumor mechanism of Naringenin, and (**D**) neuroprotective mechanism of caffeine.

The bitter taste in orange peel mainly comes from tangeretin, which is a natural bioactive ingredient [89]. According to relevant studies, tangeretin has the effect of lowering the blood glucose level and serum insulin level (Figure 4A). Specifically, it reduces the glucose tolerance test (GTT) and insulin tolerance test (ITT) values as well as clearance, thereby reducing the HOMA-IR index (an indicator of insulin resistance) [90]. As the largest endocrine organ in the human body, the liver plays a key role in insulin resistance. To further understand the action mechanism of tangeretin, researchers found that tangeretin could activate the levels of p-AKT and p-GSK 3b in isolated primary hepatocytes and mouse liver through Western blot experiments, which are signaling molecules associated with insulin sensitivity [91]. Meanwhile, tangeretin also enhances insulin sensitivity, suggesting that it has the potential to improve insulin resistance, which is an important pathogenesis of metabolic diseases such as diabetes. In addition, skeletal muscle and white adipose tissue are also important target organs for insulin action. The modulation of tangeretin in these tissues makes it a potential enhancer of insulin action. Hesperidin was also found to inhibit the p-ERK 1/2 levels in hepatocytes in a dependent manner [87]. p-ERK 1/2 is an important cellular signal transduction molecule, and its activity is closely associated with the development of insulin resistance. Previous studies have shown a negative correlation between p-ERK 1/2 activity and hepatic insulin sensitivity. When p-ERK 1/2 is activated in the liver, it leads to decreased energy expenditure, increased fasting hyperglycemia and systemic insulin resistance. To verify the role of p-ERK 1/2 in insulin resistance, researchers have inhibited p-ERK 1/2 activity by gene editing, shRNA, or specific inhibitors and found that this could improve insulin sensitivity, thus providing new strategies for the treatment of insulin resistance and type 2 diabetes. In conclusion, tangeretin, as a natural bioactive component, can reduce the blood glucose levels and serum insulin levels, and its mechanism may be related to the activation of p-AKT and p-GSK 3b levels, enhancing insulin sensitivity and inhibiting the p-ERK 1/2 levels [92].

### 4.2. Anti-Inflammatory

Oleuropein (OLP) is the main phenolic component in olive leaves and has a variety of biological activities [93]. Recent studies have illustrated that the interaction of Toll-like receptor 4 (TLR4) with hypoxia-inducible factor-1α (HIF-1α) in alveolar macrophages (AMps) plays a key role in initiating inflammatory responses during acute lung injury/reperfusion (ALI/R). TLR4 is a member of the Toll-like receptor family that can recognize various microbial products and extracellular matrix components, while HIF-1α is a transcription factor that is highly expressed in low oxygen conditions and can regulate the expression of several inflammation-related genes [94]. During ALI/R, TLR4-induced interaction with HIF-1α promotes the production of pro-inflammatory cytokines including tumor necrosis factor α (TNF-α), interleukin 1 β (IL-1β), and interleukin 6 (IL-6), which exacerbate inflammatory damage in lung tissue. The study found that OLP significantly reduced the levels of these pro-inflammatory cytokines (Figure 4B). Specifically, OLP may reduce lung I/R injury through the following mechanisms. Firstly, OLP regulates the production of TLR4-HIF-1α ring factors. It may inhibit the expression of TLR4, thereby reducing its interaction with HIF-1α and subsequently reducing the expression of pro-inflammatory cytokines. Secondly, OLP may act directly on HIF-1α to inhibit its activity, thereby reducing the expression of inflammation-related genes. Moreover, OLP may also mitigate inflammatory responses by affecting other inflammation-related signaling pathways. For example, it may reduce the production of inflammatory mediators by inhibiting the activity of nuclear factor κB (NF-κB). NF-κB is an important transcription factor that can regulate the expression of several inflammation-related genes. When OLP suppresses the activity of NF-κB, the production of inflammatory mediators is reduced, thereby alleviating the inflammatory response [95,96].

Chlorogenic acid is a dietary polyphenol compound widely found in foods, which has attracted interest for its diverse biological activities [97]. Among the numerous biological activities, the anti-inflammatory properties of chlorogenic acid are particularly striking because it has regulatory effects on the occurrence and progression of multiple diseases. HaCaT, a cell model derived from human keratinocytes, is often used to study the behavior of skin cells, which could be used to assess the anti-inflammatory effect of chlorogenic acid. In addition, primary adipocytes treated with heat-inactivated B. acnes were used to simulate the role of acne pathogens in the inflammatory process. In the experiments, these cells were treated with 10, 20, and 40 μM of chlorogenic acid to observe the effects on the levels of inflammatory cytokines TNF-α, IL-1β, IL-6, and interleukin 8 (IL-8), respectively, and examined. The results showed that the levels of inflammatory factors in these cells were significantly increased after the treatment of P. acnes, and the addition of chlorogenic acid was able to effectively neutralize this increase, indicating that chlorogenic acid has direct anti-inflammatory effects. MMPs are a class of proteases capable of resolving the extracellular matrix, and their activity plays an important role in inflammation and tumor metastasis. TLRs are a class of receptors that recognize pathogen-associated molecular patterns, which play a crucial role in initiating the immune response. The downregulation of chlorogenic acid on these molecules further reveals that its anti-inflammatory mechanisms may involve the regulation of multiple inflammatory signaling pathways. Chlorogenic acid is able to suppress the inflammatory response by reducing the activity of phosphorylated-NF-κB in HaCaT cells. This finding not only provides direct molecular evidence for the anti-inflammatory effect of chlorogenic acid, but also points to its possible route of action [98,99].

### 4.3. Antitumor

The mechanism of tumorigenesis is a complex and multifactorial process, involving the interaction of internal and external factors and the dysregulation of multiple systems in vivo. In addition to known psychiatric factors, endocrine disorders, immune deficiency, physical stimulation, and chemical stimulation, and other mechanisms exist that have not been fully revealed [100,101]. Growing evidence has highlighted the anticancer and suppressive activities of OLP, such as increasing mitochondrial apoptotic signaling, inhibiting NF-κB/cyclin D1, and reducing pAKT/pJNK/pERK1/2 signaling, have been observed in various cancer cells. Studies have shown that OLP effectively suppressed the tumor spheres and led to apoptosis via mitochondrial superoxide production and mitochondrial fragment. In vivo experiments showed that the cocoa tea extract could obviously inhibit Ehrlich ascites solid cancer. The extract of cocoa tea showed significant cytotoxic effects on the HeLa cell line, poorly differentiated nasopharyngeal carcinoma cell line, human hepatic cancer cell line, human erythroleukemia cell line, and gastric cancer cell line in vitro. Studies have shown that the EGFR signaling pathway can induce ErbB, PI3K-Akt, MAPK, and HIF-1 signaling pathways. Therefore, the inhibition of EGFR can inhibit the progression of lung cancer [102]. Naringenin has been proven to strongly bind with AKT1 and EGFR, which can regulate the EGFR-PI3K-Akt signaling pathway and ERK/MAPK signaling pathway to inhibit the production of reactive oxygen species (ROS) and the activation of NF-κB, which finally inhibits the expression of MUC5AC, thus inhibiting lung cancer (Figure 4C) [103].

### 4.4. Antibacterial

The antibacterial effect of bitter compounds is due to their intrinsic chemical structure and biological activity. Many bitter compounds contain phenols, flavonoids, alkaloids, and other components with antibacterial activity. These components are able to bind to the cell membrane or cell wall of bacteria, disrupting their integrity and leading to bacterial death. In addition, bitter compounds can also inhibit the metabolism and reproduction process of bacteria, thus playing an antibacterial role [104]. Studies have shown that bitter compounds have antibacterial effects on a variety of bacteria including Gram-positive bacteria and Gram-negative bacteria as well as certain drug-resistant bacteria [105]. For example, berberine is a common bitter compound with a broad-spectrum antibacterial effect and strong inhibitory effect on *Staphylococcus aureus* and *Escherichia coli* [106]. In addition, bitter compounds such as matrine and bitter gourd saponin also have good antibacterial activity [107,108].

### 4.5. Antioxidant

Oxidation is a common chemical process in nature that involves the transfer of electrons and the combination of oxygen atoms, which often result in changes in the properties of substances. Oxidation plays a crucial role in biological systems, from cellular respiration to energy conversion. However, oxidative effects also have two sides, and excessive or improper oxidative processes may lead to cell damage, aging, and even disease. An increasing number of studies have suggested that oxidative stress is a key factor leading to multiple chronic diseases and aging processes [109,110]. Oxidative stress refers to the imbalance between oxidation and antioxidant effects in the body, leading to the overproduction of neutral particles and reactive oxygen free radicals, which can cause damage to cells and tissues. In response to oxidative stress, the body needs to rely on antioxidants to neutralize free radicals and maintain the healthy state of cells. Free radicals are highly reactive molecules that produce oxidative stress in the human body, which damage cellular structures and damage DNA and proteins, thus triggering a variety of chronic diseases such as cardiovascular diseases, cancer, and neurodegenerative diseases. Many components of bitter compounds show significant antioxidant activity [111,112]. For example, some flavonoids, such as catechins and rutin, which are widely found in tea, red wine, and certain fruits, have potent antioxidant capacity [113]. In addition, some bitter amino acids and bitter peptides also show good antioxidant activity, and these compounds are found in both plants and animals [114].

Flavonoids demonstrate their antioxidant capacity by directly scavenging free radicals. Flavonoids are rich in phenolic hydroxyl groups, which can react with free radicals to generate stable semiquinone free radicals, thus effectively preventing the further progress of radical chain reactions [115]. Moreover, flavonoids can also exert their antioxidant effects by regulating the antioxidant enzyme system in vivo. Antioxidant enzymes are a class of enzyme that can scavenge free radicals and reduce oxidative stress. Flavonoids can bind to antioxidant enzymes and affect their configuration and activity, and thus enhance or regulate the antioxidant function of the enzymes [116]. For example, flavonoids can activate the activity of antioxidant enzymes such as superoxide dismutase (SOD) and improve their ability to scavenge free radicals, thereby protecting cells from oxidative damage [117]. In addition, flavonoids also have a role in modulating oxidative stress-related signaling pathways. Under oxidative stress conditions, a series of signaling molecules and pathways are generated in cells that play key roles in the cellular stress response and damage repair. Flavonoids can regulate the degree and progression of oxidative stress response by interacting with these signaling molecules and pathways, thereby alleviating cellular damage from oxidative stress. In conclusion, the antioxidant mechanisms of flavonoids includes the direct scavenging of free radicals, the regulation of antioxidant enzyme systems in vivo, and the regulation of oxidative stress-related signaling pathways.

### 4.6. Neuroprotective Effect

Neurodegenerative diseases are some of the most common neurological disorders such as Alzheimer’s disease (AD), Parkinson’s disease (PD), amyotrophic lateral sclerosis (ALS), and Huntington’s disease (HD). Aging is the most common factor for AD, PD, ALS, and HD, and is associated with the loss of mitochondrial function and oxidative damage caused by free radicals. In addition, aging, mitochondrial loss of function, and oxidative damage play an important role in chronic programmed cell death [118]. Caffeine is an alkaloid wilt compound, and the mechanisms by which caffeine protects nerve cells are diverse and complex (Figure 4D). Firstly, caffeine blocks the normal binding of adenine nucleoside to its recipient by binding to the adenine nucleoside acceptor. The combination of the adenine nucleoside to the receiver usually slows the activity of the nerve cells, and caffeine prevents the process and increases the activity. This increase in nerve cell activity can stimulate nerve cells to secrete hormones, such as adrenaline, which can trigger a series of physiological reactions such as a faster heart rate, increased blood pressure, and increased blood flow in the muscle. This response may help improve the ability to cope with external stimuli, thereby protecting them from injury [119]. Moreover, caffeine can also protect nerve cells by affecting adenosine receptors. Numerous studies have shown that adenosine receptors have an important role in hypoxic-ischemic diseases, and caffeine, as an adenosine receptor antagonist, can non-selectively antagonize three receptors A1, A2A, and A2B, which may play a neuroprotective role in hypoxic-ischemic events. Furthermore, caffeine also protects nerve cells by improving the release of dopamine and other neurotransmitters [120]. Dopamine is an important neurotransmitter involved in many neurophysiological processes. Caffeine enhances the release of dopamine by inhibiting the activity of adenosine receptors and thus may contribute to slowing the development of neurodegenerative disease. In addition, caffeine protects nerve cells by regulating the calcium balance. Calcium ions are crucial intracellular signaling molecules that regulate cellular functions. Excessive concentrations of calcium ions can harm and kill nerve cells. Caffeine regulates calcium ion channels, maintains calcium balance in cells, and prevents nerve cell damage from calcium overload. Moreover, caffeine can activate genes involved in nerve cell growth, repair, and regeneration. This may help restore the function of damaged nerve cells [121]. Caffeine also improves the energy metabolism of nerve cells, enhancing mitochondrial function and energy production efficiency. This supports normal nerve cell function and response to external challenges [122]. In summary, the neuroprotective mechanisms of caffeine include interactions with adenosine receptors, the modulation of neurotransmitter release, calcium ion balance, gene expression, and energy metabolism. These mechanisms contribute to protecting nerve cells and promoting nervous system health [123].

In recent years, the neuroprotective mechanism of chlorogenic acid has attracted extensive attention. Firstly, it exhibits robust antioxidant properties [124]. Oxidative stress, a significant cause of nerve cell damage and apoptosis, can be alleviated by chlorogenic acid’s ability to scavenge free radicals. This antioxidant effect preserves nerve cell function and delays neurodegenerative disease [8]. Secondly, chlorogenic acid regulates neural cell metabolism. Neural cells require significant energy for normal physiological functions. Chlorogenic acid promotes the balance of energy metabolism and enhances the energy utilization of nerve cells. It also regulates nerve cell signaling, gene expression, and protein synthesis, boosting cellular resistance [125]. Chlorogenic acid exhibits anti-inflammatory effects, reducing inflammatory factor production and the release of inflammatory factors, thereby mitigating nerve cell damage from inflammatory responses. The anti-inflammatory effect of chlorogenic acid maintains nervous system stability and health. It also improves the microenvironment of the nervous system. Chlorogenic acid can reduce glial cell activity and the toxicity of nerve cells as well as enhance nutrient supply and metabolism in nerve tissue. Chlorogenic acid protects neural cells through antioxidant, metabolism regulation, and anti-inflammatory mechanisms. These mechanisms are interrelated and interact with each other to promote the neuroprotective effect of chlorogenic acid on nerve cells. Chlorogenic acid can decrease the activity and toxicity of glial cells, or improve nerve tissue nutrition and metabolism through angiogenesis and blood perfusion regulation [126,127].

## 5. Application of Bitter Taste Compounds in the Food Industry

Foods rich in bitter ingredients are increasingly widely used in the food industry, which not only add a unique flavor to the foods, but also play an important role in nutrition and health care, medical conditioning, baking, cold drinks, and other fields. In condiments, bitter ingredients can enhance the taste of food [128]. Coffee, tea, beer, and other drinks offer a rich taste experience due to their bitter taste. Bitter spices like bitter gourd and tangerine peel are commonly used in Chinese cooking to add flavor. Bitter foods also have nutritional and health benefits. Ingredients such as flavonoids and alkaloids have antioxidant, anti-inflammatory, and antibacterial properties. Bitter foods such as bitter melon and buckwheat are popular in the health food market due to their health functions such as lowering blood sugar and regulating blood lipids. Some bitter Chinese medicinal materials, such as *coptis chinensis* and yellow cypress, are commonly used in traditional Chinese medicine for treating certain diseases. Roasters often use bitter ingredients (cocoa and coffee powder) to create diverse roasted foods. Bitter ingredients also have wide applications in cold drinks. Bitter tea and wine, for instance, are popular for their unique taste. These products are both cool and refreshing, while providing nutrients for health needs [47,83,112]. With consumers’ pursuit of healthy diets and diversified demand for food flavors, foods rich in bitter ingredients will play an important role in the fields of condiments, nutrition, healthcare, medical conditioning, baking, and cold drinks.

## 6. Challenges and Future Trends

The challenge of bitterness to human health and future trends depends on how we balance its taste and health. The metabolic process of bitter compounds in the human body is complex, and studies have shown that alkaloids (matrine) in bitter compounds could produce toxic intermediates, which may cause damage to cells. In addition, the long-term intake of excessive bitter compounds could also affect the normal function of the human digestive system, lead to indigestion, reduce appetite, and cause other symptoms [129]. More and more researchers are striving to deeply explore the biological activity of bitter compounds, action mechanisms, and their potential in the prevention and treatment of diseases. These crops will not only have a unique taste and aroma, but also provide targeted health benefits such as lowering blood pressure, regulating blood sugar, and improving cardiovascular function. Studies have shown that some bitter-taste compounds have antitumor, antibacterial, antiviral, and other biological activities, which are expected to provide new ideas and directions for the research and development of new drugs. With the development of technologies such as gene editing and biosynthesis, we can expect to develop new varieties of crops rich in specific bitter-taste compounds to meet the need for a healthy diet. Moreover, the use of bitter compounds in the food industry and drug development requires strict control to ensure the safety of products and the health of consumers. With the increasing need for personalized medicine, individualized nutritional supplements and functional foods based on bitter compounds will also attract more attention, and the food industry will also meet new challenges and opportunities, which will need to constantly innovate and improve products to meet consumers’ needs for a healthy diet.

## 7. Conclusions and Outlook

When individuals consume foods rich in bitter compounds, hTAS2Rs responds by triggering the neural pathways that lead to the bitter perception. hTAS2Rs receptors play important roles in a variety of cellular functions such as secretory action, innate immunity, and the regulation of insulin release. The role of foods rich in bitter compounds, such as bitter gourd, dandelions, *coptis chinensis*, *phellodendron chinensis*, gardenia, andrographis, purslane, dandelion, buckwheat, beans, almonds, walnuts, cashews and hazelnuts, has attracted wide attention. These bitter compounds, such as terpenoids, polyphenols, alkaloids, and amino acids, not only give food a unique taste, but also play an important bioactive role in the human body. Studies have shown that these bitter compounds, which have antioxidation, anti-inflammatory, antibacterial, and other biological activities, can effectively resist the damage of free radicals to cells, slow down the cellular aging process, and have significant benefits in preventing a variety of chronic diseases such as cardiovascular disease, cancer, and diabetes. Fully characterizing the physiological and pathologic properties of hTAS2Rs in humans will provide insights into the health effects of bitter compounds on humans. Whether the health benefits produced by these bitter compounds are related to the binding of other receptors or channels also needs further study. In addition, how to control or reduce the toxic intermediates produced by bitter compounds is also a hot topic of concern.

## Figures and Tables

**Table 1:** The sources of dietary caffeine in people’s daily life.

Sources of Caffeine	Mean Concentration
Espresso	143 mg/43 mL
Coffee	72.8 mg/125 mL
Tea	13.8 mg/120 mL
Chicory beverage	16.1 mg/125 mL
Cola	17.6 mg/330 mL
Chocolate candy	2.1 mg/30 g
Plain chocolate	17.8 mg/30 g

## Data Availability

The original contributions presented in this study are included in the article. Further inquiries can be directed to the corresponding author.
